# Customisable X-ray fluorescence photodetector with submicron sensitivity using a ring array of silicon p-i-n diodes

**DOI:** 10.1038/s41598-018-33966-y

**Published:** 2018-10-29

**Authors:** Phil S. Yoon

**Affiliations:** Brookhaven National Laboratory, P.O. Box 5000, Upton, NY 11973-5000 USA

## Abstract

The research and development of silicon-based *X*-ray fluorescence detectors achieved its submicron sensitivity. Its initial use is intended for *in-situ* beam monitoring at advanced light-source facilities. The effectively functioning prototype fully leveraged technologies and techniques from a wide array of scientific disciplines: *X*-ray fluorescence technique, photon scattering and spectroscopy, astronomical photometry, semiconductor physics, materials science, microelectronics, analytical and numerical modelling, and high-performance computing. At the design stage, the systematic two-track approach was taken with the aim of attaining its submicron sensitivity: Firstly, the novel parametric method, devised for system-wide full optimisation, led to a considerable increase in detector’s total solid angle (0.9 steradian), or integrated field-of-view (~3000 deg^2^), thus, in turn, yielding a substantial enhancement of its photon-detection efficiency. Secondly, the minimisation of all types of limiting noise sources identified resulted in a boost to detector’s signal-to-noise ratio, thereby achieving its targeted range of sensitivity. The subsequent synchrotron-radiation experiment with this *X*-ray detector demonstrated its capability to respond to 8-keV photon beams with 600-nanometre sensitivity. This Article reports on the innovative and effective design methods, formulated for systematising the process of custom-building ultrasensitive photodetectors, and future directions.

## Introduction

**Figure 1:**
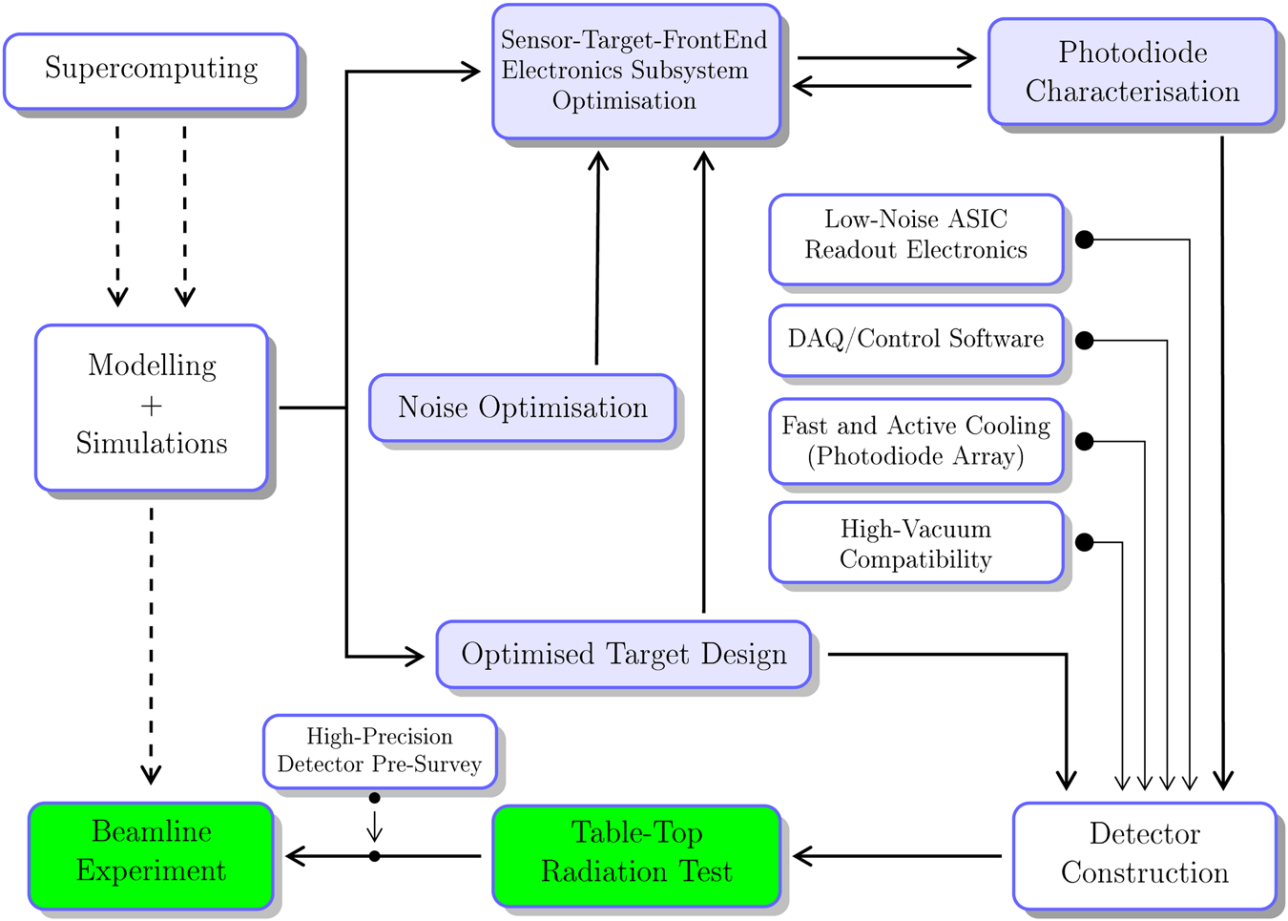
Prototyping pipeline for the detector development. Block diagram illustrating the workflow of the *X*-ray detector development; shown in blue boxes is the four-phase design stage of optimising the sensor-target-frontend subsystems. The calculation results from analytical modelling and supercomputer-aided Monte-Carlo simulations provided quantitative and practical guidance (dashed line) on conducting time-efficient beamline experiments.

Contemporary synchrotron-based light-source facilities worldwide are placing greater and greater user demand for *in-situ* instruments having ultrahigh spatial sensitivity. The purpose of utilising such an ultrahigh-precision instrument is to fully realise the benefits of higher brightness and minuscule dimensions of *X*-ray beams radiating from a light source. To this end, numerous groups^1–8^ have developed *X*-ray detectors of this type to fulfil their own needs over the past decades. In the meantime, the advent of new synchrotron-radiation (SR) facilities of this decade^9,10^ is sparking a strong need to further increase photodetector’s sensitivity to a scale of a fraction of one micron. Driven by such growing needs for ultrahigh-precision beam-monitoring devices, intensive R&D efforts were dedicated to developing hard/tender *X*-ray detectors capable of monitoring nanometre-size photon beams *in situ*. Apart from the pinpoint spatial sensitivity, the *X*-ray fluorescence (XRF)^11,12^ technique was utilised, by design, for the semiconductor-based detector. Its initial applications are intended for downstream photon-intensive experiments. Using this photodetector of submicron sensitivity, *X*-ray beams focused onto beam-defining optical slits can be kept on the upstream side of minute biological samples under study (Fig. 1).

At the inception of this R&D project, a handful of design considerations were taken into account and thus led to building effectively functioning prototypes. The principal design goals pursued are listed in order of priority as follows: (1) submicron-scale sensitivity, (2) preservation of *X*-ray beam properties, such as photon flux and relative energy spread (Δ*E*/*E*), on the downstream side of the detector, (3) ultralow-noise operation, (4) high-vacuum compatibility, and (5) compact and cost-effective design. As identified, these five considerations are foundational for the design of position-sensitive detectors that are highly efficient for detecting photons^13^. The model-based full optimisations, based on which the detector prototype was constructed, gave the design a boost to its photon-detection efficiency and enabled a resulting enhancement of signals. In parallel, active and fast detector-cooling modules and control system for photon-counting application were integrated into the low-noise detection system. Consequently, it was made possible to create a novel avenue towards a boost to detector’s signal-to-noise (SNR) ratio, and a pathway of achieving its ultrahigh spatial sensitivity. Additionally, utilising the parametric optimisation method can empower detector designers to nimbly react to satisfy varying needs for individual beamline programs at light-source facilities. Illustrated as a whole in Fig. 1 is the codified process of prototyping a custom detector attaining the targeted sensitivity.

## Results

### Principle of operation

**Figure 2:**
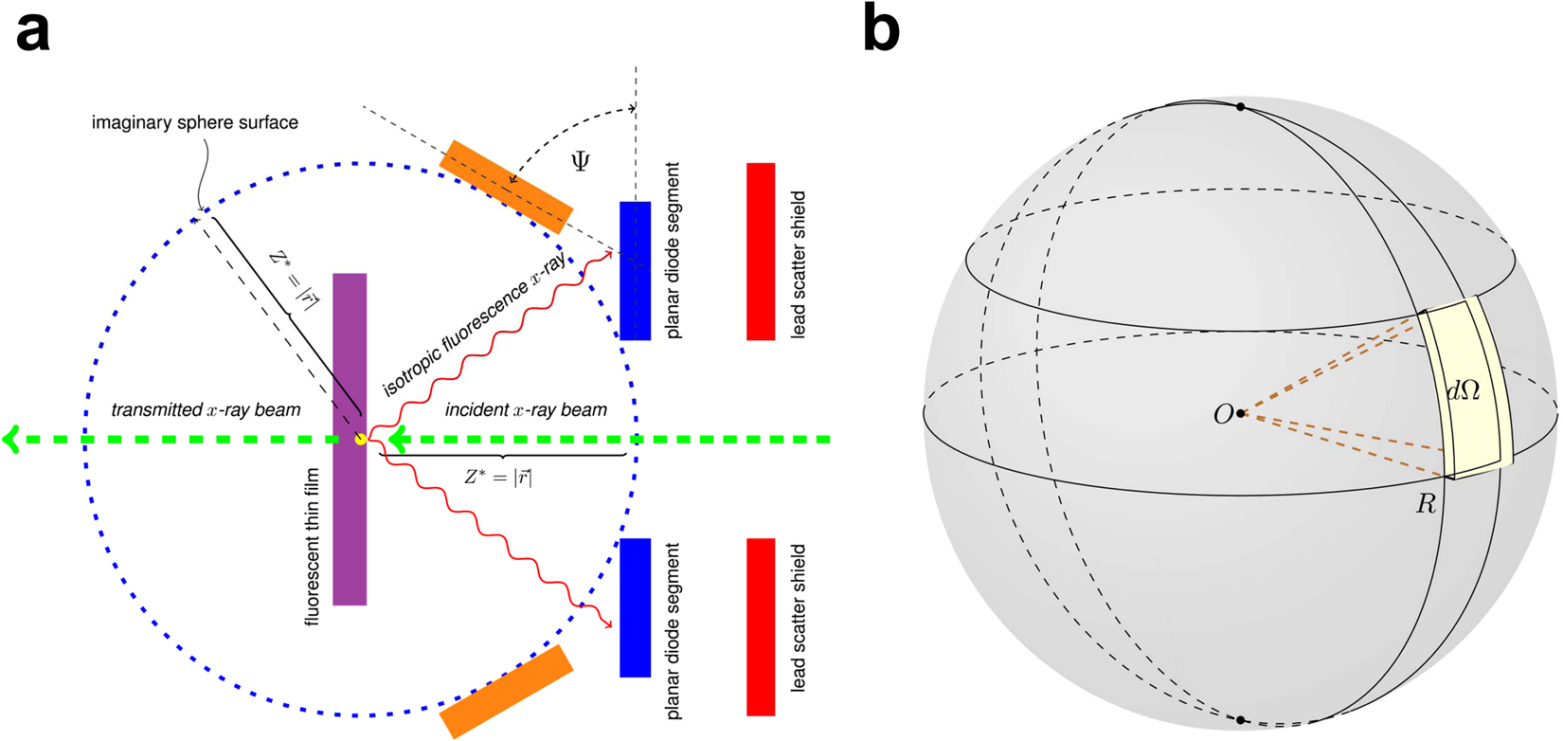
Principle of detector operation. (**a**) Schematic presentation of the operational principle of the *X*-ray fluorescence detector. Drawn with the blue dashed line is the cutaway view of solid angle’s imaginary sphere having its radius equal to the working distance Z^*^. The projection angle Ψ is formed between the sensor segment (solid blue) in the transverse plane and the curvilinear sensor (solid orange) tangential to the spherical surface; note that the thin film, diode segments, and lead shield shavings are not to scale. (**b**) Solid angle, subtended at a secondary radiation source on the thin film, is defined on the surface of the imaginary sphere.

As illustrated in Fig. 2(a), the detector system design consists of four key functional components: (1) a ring array of *Si* p-i-n diodes serving as photosensors, (2) a thin metallized film as a source of fluorescence radiation, (3) its dedicated ASIC-based readout electronics, and (4) micro-thermoelectric (Peltier) pads and a water-cooling module, all of which are interfaced with a copper heat sink, or heat exchanger. In essence, the sensor-target subsystems, surrounded by the readout electronics and cooling modules, are situated at the very heart of the detector system. Importantly, the thin fluorescing film–henceforth referred to as fluo-film, or fluo-target, or target for short–is introduced to the design in order to act as a source of secondary *X*-ray radiation emitted upon impingement of primary *X*-ray radiation. As an addition, a lead (Pb) scatter shield, or visor, is inserted immediately upstream from the sensor array so as to safeguard the ultrasensitive photosensors against potential stray signals that may arise during operation. And both upstream and downstream beryllium windows on a multi-port vacuum chamber are designed to be thin enough in consideration for downstream photon-starving experiments.

**Figure 3:**
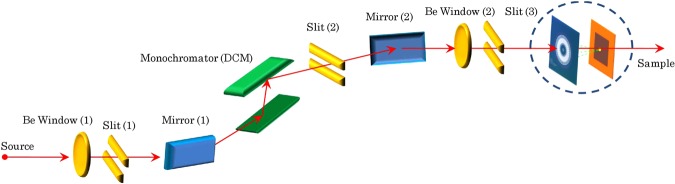
Beamline layout. An envisioned beamline layout is illustrated with the inclusion of the *X*-ray fluorescence detector installed downstream from a string of optical components (i.e., a double-crystal monochromator (DCM), mirrors, beam-shaping pre-slits, and an exit-slit collimator). One representative unit is shown to be positioned upstream from a sample.

As depicted in Fig. 2(b), the effective beam-through apertures are set to accept, at least, a 5-*σ* footprint of an incident beam. The dimension of this central aperture, accordingly, limits the linear working range to a few millimetres for a highly focused nanometre-size beam, based on the 0.5-*μ* rad tolerance for the beam-pointing stability required by the National Synchrotron Light Source II (NSLS-II) facility^10^. Focused *X*-ray beams are then guided to propagate unimpeded in a high vacuum, passing through the circular apertures on both the sensor ring and the scatter shield. Moreover, the fluorescing target is arranged in a configuration orthogonal to the propagating direction of incident *X*-ray radiation. When primary radiation impinges upon the fluo-film, a paucity of *X*-ray radiation^11^ emanates in the backward direction in the form of characteristic *X*-ray emission. As a result, the emission of nascent fluorescence radiation illuminates the backside of the sensor array. At the same time, the vast majority of incident photons are transmitted. In this process, the orthogonal beam-target topology becomes a critical factor for monitoring a monochromatised beam with ultrahigh precision. On this account, the orthogonal target configuration is required for three main reasons: First, the normal incidence forces an incident beam to experience from beam’s vantage point an uniform effective thickness of each fluo-target, regardless of its point of incidence. Thus, the normal incidence of primary radiation on the target makes it possible to preserve the incident beam properties during the process of transmission and propagation towards a sample at the opposite end of the beamline from the light source. Second, the normal incidence on the target, in turn, ensures isotropic illumination and uniform photon detection over the entire active area of the planar sensor array. And third, during transmission of *X*-ray beams through the thin film, scientists can derive its beam centroid from its entire beam profile, benefiting from uniform illumination over the diode array. On top of the orthogonal configuration, suppression of background-event signals is another critical issue. Taken all together, the backward-scattering mode of operation was favoured over the forward-scattering mode in the light of minimising potential systematic uncertainties arising during operation. The choice of the operation mode was borne out by the fact that the former mode can suppress elastically scattered photons (i.e., Rayleigh scatterings) coming from the target. Consequently, the sensor array captures backward-scattered fluorescent radiation with higher detection efficiency and spectral purity. Furthermore, three decisive advantages of using secondary *K*_*α*_-fluorescent radiation are the following^14^: (1) distinctively high intensity and variable photon flux, (2) high spectral purity, and (3) the availability of a wide selection of fluo-film materials with reference to the energy of incident radiation. To visualise its full range of beamline operation, Fig. 3 shows a schematic setup for monochromatic beamline components and the *X*-ray detector. One of the design features of note is that its compact lateral form factor (<1 ft.) makes it possible for each beamline to accommodate multiple colocated detectors. This way a set of *X*-ray detectors operational along a beamlne enables extracting more beam parameters, such as divergence angles, beam emittances, and so forth.

### Photosensor design

As an optical receiver, the photodiode ring array is coupled with a fluo-target and its dedicated front-end readout circuitry, forming the trio core subsystems. It is thus of foremost importance for low-noise signal processing to design a sensor ring array that is most optimal for both the target and the readout electronics.

**Figure 4:**
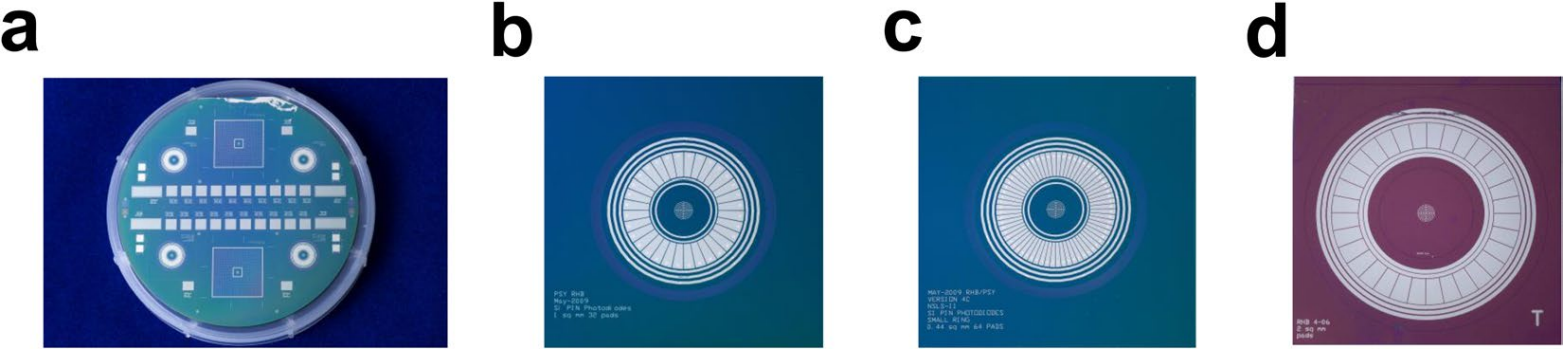
Prototype sensors. (**a**) A finished *Si* wafer containing four 32-segment diode rings arranged in each quadrant. (**b**) [Prototype-II-32] A fully optimised layout of the photodiode ring array composed of 32 segments, each of which having its surface area of 1.0 *mm*^2^. (**c**) [Prototype-II-64] A ring array of the 64 diode segments, each measuring 0.44 *mm*^2^. (**d**) [Prototype-I-32] An unoptimised version of the 32-segment diode array.

**Figure 5:**
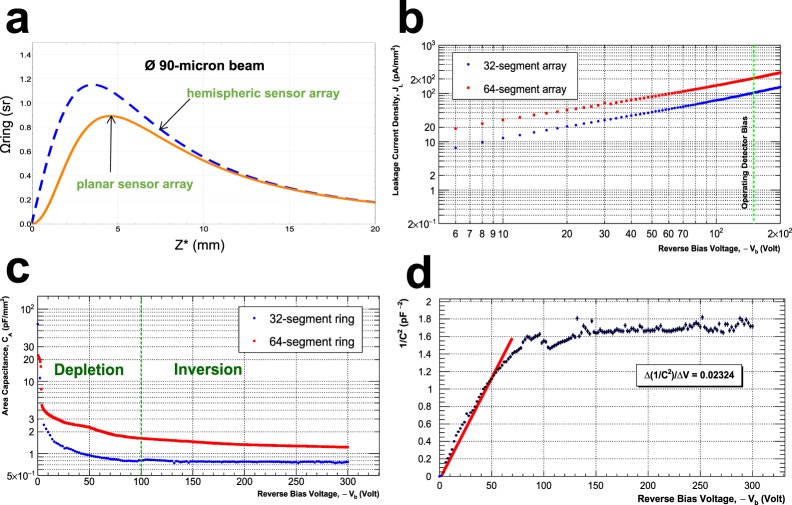
Sensor characterisation. (**a**) [Prototype-II-32] Profiles of the total solid angles Ω_*ring*_ over working distance Z*. The lower trace (red solid) corresponds to the optimised planar sensor array Ω_*ring*_, and the upper trace (blue dashed) to the optimised hemispheric array of sensors Ω_*hemi*_. (**b**) [Prototype-II-32/-64] Measured *J*_*r*_ − *V*_*r*_ characteristics of the photodiode segments at ~300 K and 1 atm.; the blue trace corresponds to the 32-segment ring and the red trace to the 64-segment ring. Under the reverse-biased DC voltage across the device up to 200 V, leakage current densities *J*_*r*_ of the 32-segment and 64-segment ring array increase at ~300 K. The vertical green dashed line marks the operating detector bias of −150 V. (**c**) [Prototype-II-32] Measured *C*_*A*_ − *V*_*r*_ characteristics of the *Si* p-i-n diode segment at ~300 K and 1 atm.; plotted against the reverse DC bias on a log scale is the areal capacitance undergoing the depletion and the inversion processes. The upper trace in red corresponds to the 64-segment array and the lower trace in blue to the 32-segment array. (**d**) [Prototype-II-32] Plot of $\mathrm{1/}{C}_{A}^{2}$ as a function of reverse-biased DC sweep −*V*_*r*_ showing the dopant concentration of the semiconductor junction device. A linear fit (red) is applied to the data points with systematic errors propagated in the depletion region.

Achieving the targeted sensitivity level presents two main challenges to the design: (1) how to give rise to a substantial increase in photon-detection efficiency and (2) how to realise ultralow-noise photon-detection and operation under a high photon flux (>10^13^ photons/s) environment. The two-track approach was correspondingly taken to rise to the said challenges: The first approach was to develop an effectual method of enhancing a higher degree of photon-detection efficiency, resulting in acquiring far more signals from the sensor array. Unlike the prior developments elsewhere^1–5^, the sensor designs take the form of a multisegmented annular ring, covering the full range of 2*π*-azimuthal angle, thus enabling highly efficient capture of isotropic fluorescent radiation (Fig. 4(a–d)). As the first step towards the photometric optimisation, calculations of solid angle Ω_*ring*_^15^, subtended by the diode ring array at a source of fluorescence radiation, were worked out by virtue of finding real-valued analytical functions (Eq. (S1 and S2)). Referring to Fig. 2(b), the four-parameter solid angles, subtended at a point source, are expressed by Eq. (1a),(b). Here, Ω_*ring*,4_ and ${\tilde{{\rm{\Omega }}}}_{ring\mathrm{,4}}$ denote respectively a solid angle for the planar sensor and a solid angle projected onto the sphere surface.

1a $${{\rm{\Omega }}}_{ring,4}=\mathrm{\ 2}{N}_{s}\{\arctan (\frac{{\theta }^{+}{z}^{\ast }}{\sqrt{{({\rho }^{-})}^{2}+{({\rho }^{-}{\theta }^{+})}^{2}+{({z}^{\ast })}^{2}}})-\arctan (\frac{{\theta }^{+}{z}^{\ast }}{\sqrt{{({\rho }^{+})}^{2}+{({\rho }^{+}{\theta }^{+})}^{2}+{({z}^{\ast })}^{2}}})\}$$

1b $${\tilde{{\rm{\Omega }}}}_{ring\mathrm{,4}}({z}^{\ast }\,;{\theta }^{+},{\rho }^{+},{\rho }^{-})=Pr(\psi )\cdot {{\rm{\Omega }}}_{ring,4},$$

where the projection factor Pr(*ψ*) is defined as cos*ψ* (Fig. 2(a)). The variables z^*^ and *N*_*s*_ represent respectively the working distance of the target from the sensor plane and the total number of diode segments in the array. And *θ*^+^, *ρ*^+^, and *ρ*^−^ signify polar angle, radial coordinate components of the sidelines of each diode segment, respectively (Supplementary Fig. 1). In the case of a planar sensor (Fig. 2(a)), the projection factor Pr(*ψ*) comes into play when calculating solid angles with precision. First and foremost, the application of this effectful parametric method allows quantitative determination of the working distance optimal for the target. As observed in Fig. 5(a), the optimised sensor array attains its total peak solid angle of 0.90 steradian, or integrated field of view of ~3000 *deg*^2^, under irradiation with photon beams having a 90-*μ*m diameter at an optimal working distance of 4.5 mm–the number of photon counts peaks out when target’s working distance is at this calculated optimum position. Given these parameters, the photon-detection efficiency *η*_*det*_ of the optimised core subsystems is estimated to be ${\eta }_{det}={{\rm{\Omega }}}_{ring}\mathrm{/4}\pi ={\sum }_{id=1}^{{N}_{d}}\,{{\rm{\Omega }}}_{id}\mathrm{/4}\pi =0.072$. Ultimately, this parametric method provides a quantitative basis for determining the optimal layout and dimensions of the sensor array with the inclusion of multiple guard rings and the central aperture. The foregoing analytical model made it possible to perform a series of rigorous analyses for fine-tuning the optimisation of the core subsystem. According to the calculation results, two optimised versions were fabricated—dubbed Prototype-II-32 and Prototype-II-64—in reference to the unoptimised Prototype-I. As Fig. 4(b–d) shows, the total surface areas of Prototype-II were halved, whereas its peak solid angle and photon-detection efficiency doubled as a direct result of the photometric optimisation. In addition, the full optimisation, by including spatial constraints imposed by indirect-bandgap semiconductors, produced a compact design, thus creating enough room for multiple circular-concentric guard rings. Accordingly, a system of two inner and three outer guard rings was incorporated in order to step smoothly down the electric potential to the ground (Fig. 4(b,c)). As a side benefit, the resulting miniaturisation made its way for lower-cost and higher-volume manufacturability on a single wafer. And the second approach was the minimisations of the system-wide noise levels achieved in various ways. As part of the optimisation process, the idea of multisegmentation was introduced to the annular diode design. It was based on the fact that the multisegmentation design offers a few benefits to recognise: In the first place, the multisegmentation allows for the reduction in junction capacitance, thereby suppressing series noise and enabling faster response. In the second place, this multisegmentation provides the benefit of creating fine-tuning knobs for adjusting diode capacitance to match with the front-end input capacitance. Upon sensor fabrication, electrical characterisation was performed on individual diode segments. Follow-up extraction of the characteristic parameters in detail rendered quantification of detector’s performance, which was fed into the optimisation loop, as shown in Fig. 1. When irradiated with photon beams of 90-*μm* diameter, the total solid angle of the planar sensor array peaks at 4.5 mm, whereas an optimised hemispheric sensor array is expected to bring about 30% increase in its solid angle (Fig. 5(a)). It was observed from Fig. 5(b) that surface leakage current density *J*_*r*_ linearly increases with the reverse-biased voltage applied across the p-i-n junction with ohmic contacts. In particular, the *C*_*A*_ − *V*_*r*_ measurements took into account leakage currents as a dissipation factor (Fig. 5(c)). Furthermore, dopant concentration was obtained from the slope of the $\mathrm{1/}{C}_{A}^{2}-{V}_{r}$ curve in the depletion region (Fig. 5(d)). And it was ascertained from Fig. 5(b) that the level of leakage current density *J*_*r*_ is held below 100 *pA*/*mm*^2^ under reverse-biased DC sweep down to −150 *V* at room temperature, while satisfying a set of the design criteria. On the other hand, under the applied electric field, sensor capacitance decreases until full depletion is reached at around −150 *V* (Fig. 5(c)). In the inversion region, the areal capacitance *C*_*A*_ as low as 0.5 pF was measured at the maximum depletion depth on 100 *kHz*, based upon which the capacitive matching was conducted (Fig. 5(c))^16,17^. Listed as a summary in Table 1 are salient detector parameters extracted from the *J*_*r*_ − *V*_*r*_ and *C*_*A*_ − *V*_*r*_ measurements. A silicon p-i-n diode of high-speed response produces sufficiently low leakage currents, even at room temperature, without requiring a cryogenic cooling system. Moreover, a wide dynamic range and excellent linearity are inherent to the silicon material. Cognisant of these properties, silicon was opted, over other semiconducting materials, for the detection of hard/tender *X*-ray radiation (2~25 keV). Notably, the sensor array operates as a double-side junction–the frontside (device side) and the backside (window side)-illuminated structure^18,19^.

**Table 1:** Figures of merit extracted for the array of 32-segment *Si* diodes; unless otherwise stated, each parameter was obtained from *J*_*r*_ − *V*_*r*_ and *C*_*A*_ − *V*_*r*_ measurements taken at ~300 K and 1 atm with the reverse bias of 150 V and of an AC signal at 100 kHz. Not*e* that *e* and *h* denote electron and hole, respectively.

Parameters	Values
Total sensor thickness, *T*	480 *μ*m
Silicon oxide thickness, *T*_*ox*_	560 nm
Depletion voltage, *V*_*d*_	~150 *V*
Depletion depth, *W*_*d*_	200 *μ*m
Leakage current density, *J*_*r*_	0.12 [260 K]/104 [300 K] *pA*/*mm*^2^
Effective dopant concentration, *N*_*eff*_	5.0 × 10^12^ *cm*^−3^
Resistivity, *ρ*	1.0 *k*Ω-*cm*
Extrinsic Debye length, *L*_*d*_	1.85 *μ*m
Junction capacitance, *C*_*j*_	0.5 *pF*
Shunt resistance, *R*_*sh*_	180 *G*Ω
Carrier transit time, *t*_*tr*_	10.2 [*e*]/34.1 [*h*] *ns*
Rise time response, *t*_*r*_	1.9 *ns*
RC time constant, *τ*_*RC*_	0.9 *ns*
Time resolution, *τ*_*r*_	2.8 *ns*
Bandwidth, *f*_*BW*_	180 *MHz*
Noise equivalent power (*λ* = 229 nm)	1.6 × 10^−11^ $W/\sqrt{Hz}$
Specific detectivity, D*	6.4 × 10^−10^ $mm\sqrt{Hz}/W$

### Thin fluorescing film

**Figure 6:**
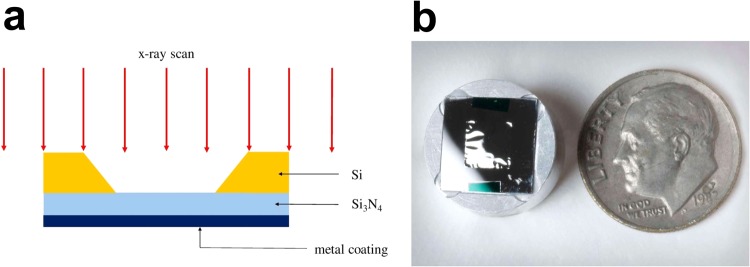
Fluorescing target. (**a**) Cutaway view of the chromium (Cr) film vacuum-sputtered on the *Si*_3_*N*_4_ substrate window. (**b**) Image of a fluorescing film mounted on an aluminium placeholder is shown with a US dime coin for comparison of dimensions.

A high-transmission fluo-film optimal for the incident beam energy was constructed with the identification of the following elements to consider: (1) the range of the energy of the *X*-ray beam in operation, (2) the optimal range of beam energy to which the detector responds, (3) K-shell absorption edge of the fluorescing target material, and (4) the intensity expected in the *X*-ray beam. A priori information described above sets standards for selecting appropriate film materials. Hence, the selection of materials becomes a necessity, based on their K-shell values having sufficient separation below the energy of incident *X*-ray beams. The following is a list of the selection along with respective values of *K*_*α*_ and wavelength *λ*: _22_*Ti* (*K*_*α*_ = 4.511 keV; *λ* = 2.749 *Å*), _24_*Cr* (*K*_*α*_ = 5.415 keV; *λ* = 2.290 *Å*), _25_*Mn* (*K*_*α*_ = 5.899 keV; *λ* = 2.102 *Å*), _26_*Fe* (*K*_*α*_ = 6.404 keV; *λ* = 1.936 *Å*), _27_*Co* (*K*_*α*_ = 6.930 keV; *λ* = 1.789 *Å*). The *X*-ray transmission efficiency and fluorescence signals contend with each other. The optimal thickness of each of the film materials is determined from the values of mass-absorption coefficients $\frac{\mu }{\rho }$ presented in the literature^20,21^ and numerical simulations that follow (Supplementary Fig. 2). In such a way, transmission rates of incident photon energy of 8-keV are above 90% with a selection of differing metallic media for use as a thin film. The transmission efficiency of an incident *x*-ray beam is particularly important for photon-starving experiments being conducted at synchrotron light sources. The thickness of thin metal film is often too feeble to enable a self-supporting fluo-target of adequate quality. As a solution, a robust *X*-ray transmission window was introduced to the fluo-film design. The substrate material of choice is the commercial silicon nitride (*Si*_3_*N*_4_) window (Norcada NX10500E, Supplementary Fig. 3), which is known to be a low-stress (<250 MPa) radiation-hard substrate for *X*-ray applications^22,23^. For providing sufficient mechanical strength, a 500-nm thick single-layered film was vacuum-sputtered through a mask onto the silicon-nitride window (5 mm × 5 mm × 500 nm) at the NSLS-II in-house facility^24^. Displayed in Fig. 6(a,b) are the structure of the *X*-ray window and a thin film supported by the window substrate mounted onto an aluminium placeholder.

### Photon-transport Monte-Carlo simulations

During detector operation, background signals from coherent elastic and incoherent inelastic scattering may be emitted anisotropically in addition to fluorescent signals, owing to the polarisation of synchrotron radiation in the plane of synchrotron^25,26^. For the quantitative assessment of signals and backgrounds, Monte-Carlo (MC) simulation techniques were employed to study *X*-ray absorption, scattering, and fluorescent radiation. Relying on the use of EGS4-based RÖntgen SImulator (RÖSI version 0.19) package^27,28^, the MC simulations were conducted with the inclusion of the geometry of the entire detector assembly and the vacuum chamber. Detector simulations, using realistic statistics of photon flux of the order of 10^12^ photons/s, were carried out on high-performance supercomputing platforms^29^. The primary purposes of such high-fidelity MC simulations are the following: (1) quantification analysis of detector performance and (2) estimation of potential background events coming from ambient scatterings, both of which are important for attaining its submicron-scale sensitivity. This MC model collectively included signals from *X*-ray fluorescence and Rayleigh and Compton scatterings. In particular, the angular distribution of Rayleigh photons of a few keV, which are scattered from medium- or high-Z materials, is confined mostly in a wide-angle cone open in the forward direction^30,31^. For the photon energy ranging from 10 to 50 keV, the photoelectric interaction is the dominant signal process, having sole responsibility for the fluorescence phenomenon. On the other hand, Rayleigh scattering is the dominant background process over Compton scattering in the energy range of 5~25 keV. With the selection of materials (i.e., _24_*Cr*, _25_*Mn*, _26_*Fe*, and _27_*Co*), MC studies show that estimated contributions from the two competing interactions–Rayleigh and Compton scatterings–are as insubstantial as below the level of 1.0% and 0.1%, respectively. As defined in Eq. (2), the photon purity *P*_*K*,*fluo*_ of the K-shell fluorescent radiation is the ratio of K-shell fluorescent radiation *N*_*K*,*fluo*_ to a sum of scattered radiation *N*_*scatt*_ and *N*_*K*,*fluo*_:

2 $${P}_{K,fluo}={N}_{K,fluo}/({N}_{K,fluo}+{N}_{scatt})$$

Further, the MC simulations indicated that the purity *P*_*K*,*fluo*_ for each individual element in the selection amounts to nearly 100% in a vacuum.

### Readout electronics and peripheries

**Figure 7:**
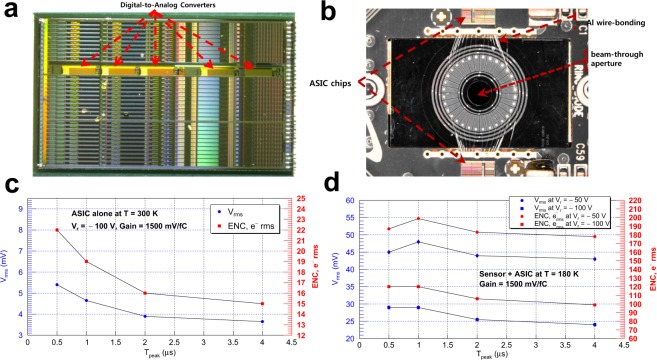
Sensor-ASIC interconnections and front-end readout noise performance. (**a**) Image of the High-Energy Resolution Multi-Element Spectrometer (HERMES4) ASIC chip of which layout dimension is 3711 *μ*m × 6287 *μ*m. (**b**) Details of the sensor-ASIC layout displaying on-chip interconnections. (**c**) Analysed is noise originated from the HERMES4 ASIC chip alone without photogenerated current at 300 K. The blue circles in the plot represent rms voltages in units of mV, while the red squares do the measured equivalent noise charge (*e*^−^ rms) at different selections of peaking time *T*_*peak*_ (0.5, 1, 2, and 4 *μ*s). Note that the gain is set to 1500 mV/fC. (**d**) Plotted as a function of *T*_*peak*_ are the rms voltages arising from the sensor array wire-bonded to the two ASIC chips at near cryogenic temperature (~180 K).

Compact in-vacuum readout electronics were designed, utilising dedicated application-specific integrated circuits (ASICs) for the high-rate photon-counting application. The HERMES4 ASIC, based upon 350-nm CMOS technology, offers 32 channels of low-noise charge amplification, high-order shaping with baseline stabilisation, and peak detection for various low-noise analogue/digital processing (Fig. 7(a))^32,33^. For reducing stray capacitance and for suppressing signals stemming from electrical interconnections, each sensor segment in the array is directly wire-bonded to the input of the front-end readout channel (Fig. 7(b)). As such, Al-wire wedge bonding and the ultrasonic technique were utilised between the input of an ASIC channel and a bonding pad implemented on each diode segment. According to the measurements, ASIC noise optimisation enables an electronic resolution of 15 *e*^−^ rms (equivalent noise charge, or ENC) with a choice of 4-*μ*s peaking time at room temperature. With multisegmented photosensor’s high finesse, the event rate per each ASIC channel is diminished to 40 kHz in a high-flux SR condition. As a result, the front-end readout with low-power consumption (8 mW/channel) was realised. For high-flux applications, one important consideration is how to design associated readout electronics capable of processing sufficiently high photon statistics without reaching charge saturation. It was observed during a beamline experiment that the processing rate of this ASIC readout chip can cope with ~100 kHz per channel. Additionally, the integrated cooling subsystem functions to lower the operating temperature of the *Si* diode array nestled on the readout printed circuit board. Hence, the active and fast detector cooling is an added functional feature that helps minimise persistent system-wide parallel noise. In this respect, efficient cooling modules are integrated into the design in three ways: (1) A ring array of thermoelectrically-cooled diodes is included in the power budget for minimising bulk leakage currents. (2) Both the readout electronics and the thin film make exceptionally good thermal contact with a water-cooled heat sink copper block. And, (3) applying adhesive with high thermal conductivity (5.77 W/m K) for interconnecting individual components yields a remarkable enhancement of the heat-transfer process. As a consequence, its operating temperature can plummet to −40 °C in as fast as a few seconds after power-up. As an integral component of the detector system, the mechanical support made of an Invar 36 (Alloy 36)^34^ was designed together. This instrument support system of high thermal stability (100 nm/°C/hr) ensures the steady maintenance of detector’s high-precision alignment and spatial sensitivity in a temperature-controlled environment throughout each year.

### Radiation experimentation

**Figure 8:**
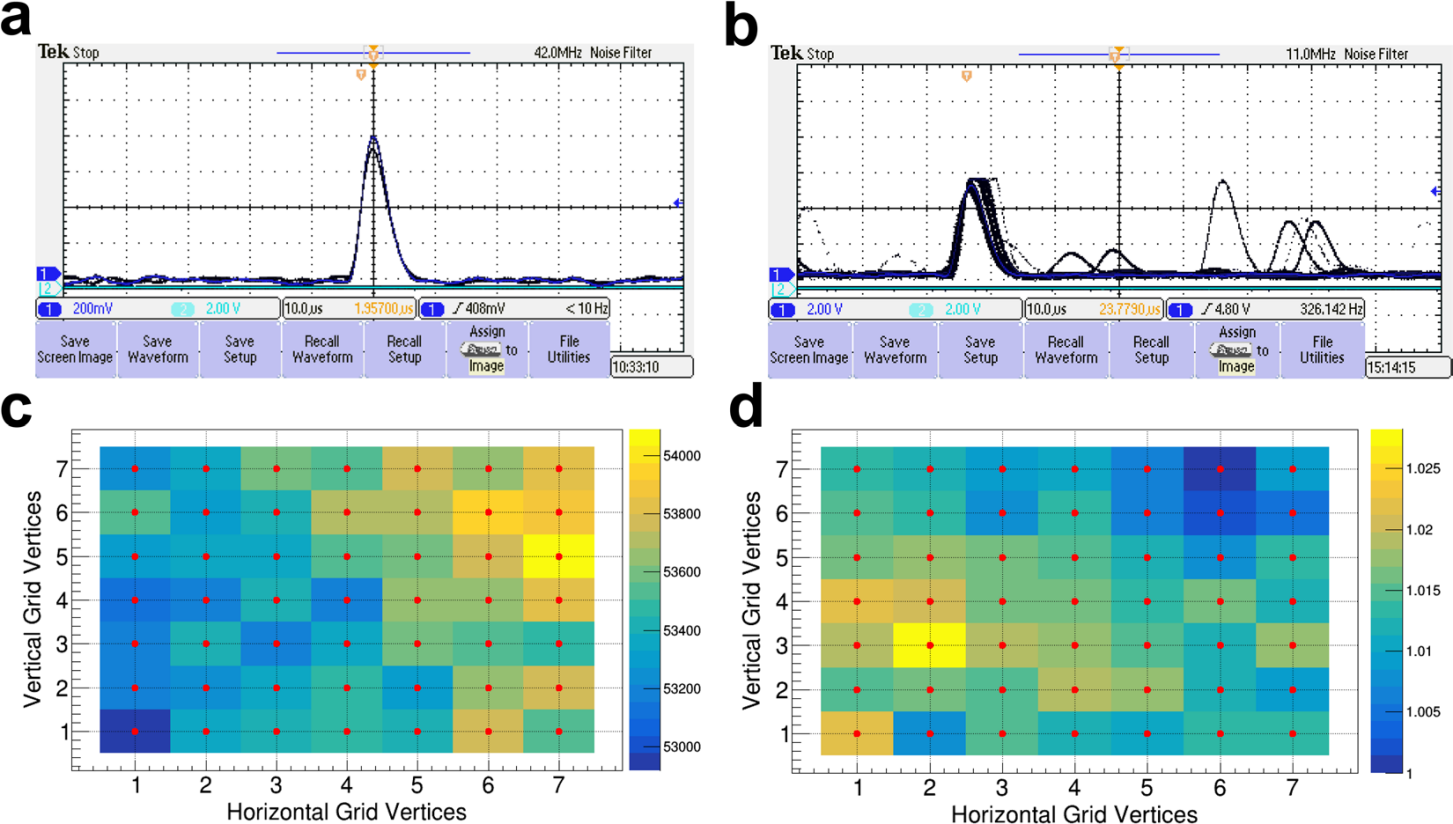
Beamline experimentation. Real-time images of pulse-shaped output waveforms captured during detector operation at −40 °C, (**a**) when irradiated with 5.9-keV radiations from Fe-55 sealed sources in a high-vacuum condition at the base pressure of 10^−4^ torr, and (**b**) when irradiated by a synchrotron radiation in a high-vacuum condition of 3 × 10^−6^ torr. (**c**) Mesh plot showing photon counts processed from the ASIC channel 34 at each vertex (red dot) over the 7 × 7 mesh grid. The variations of photon counts projected over the mesh grid are mapped to the colour scales on the right-hand column. (**d**) Photon counts processed from the channel 46 are normalised to the minimum count at each vertex and are then projected onto the same 7 × 7 mesh grid.

As a proof of concept, a table-top experiment was carried out first with an Fe-55 sealed radioactive source, confirming the acquisition of noise-free detector response (Fig. 8(a)). Next, the prototype detector, loaded with the fully optimised 32-segment sensor (Prototype-II-32) and target, was experimented with polychromatic beams at the X7A beamline of the National Synchrotron Light Source. Prior to the SR experiment, high-precision alignment (*σ* < 60 *μm*) of the detector assembly was ensured first at the beamline in an effort to minimise systematic uncertainties (Fig. 8). The amount of detector noise, intrinsic to the radiation sensor and the front-end readout system, was consistently minimised during both the table-top and the SR beamline experiment. As consistently evidenced by the experimental observations, the noise floor was brought down to the bottom level at −40 °C in high-vacuum conditions (Fig. 8(a,b)). Then, a subsequent SR experiment was dedicated to one full round of beam-based transverse aperture scan with *X*-ray beams of 90 *μm* in diameter in regular steps of 600 nanometres (nm) over 7 × 7 mesh grid. All the photon counts, registered by individual diodes during the aperture scan, are represented with corresponding colour codes (Fig. 8(c,d)). Each colour map, projected over the mesh grid, indicates the mapping of photon counts into the colour scales. This way the mesh plots visualise the variations of photon counts at each of the 49 vertices with the submicron interval. Hence, these colour maps clearly delineate that the detector design has its capability to respond with 600-nm sensitivity and better under intense irradiation of *X*-ray photon beams at the given energy.

## Discussion

Leveraging the systematic design method, developed for fully optimising the detector system and for eliminating noise sources, maximised its SNR and thus boosted its spatial sensitivity. Consequently, the long-standing barrier of detector’s submicron sensitivity has been broken down. This breakthrough was achieved by amalgamating technologies from manifold areas of research fields. Eventually, the custom-design method, offering great flexibility to add more new ideas and features, became a product of the transdisciplinary approach, thereby ushering in the realm of nanometre-range sensitivity. In a conventional prototyping process, a significant amount of lead time and cost is needed for completing an entire detector prototyping process. By contrast, taking the two-prong approach reported in this Article makes it possible to design an archetype of the photodetector reaching a desired level of sensitivity at a fraction of the previously required time and cost. Above all, the detector design, ensuring normal incidence of primary radiation, is particularly apposite to highly focused monochromatised *X*-ray beams, expecting its surpassing photometric performance. Further enhancements of its detection efficiency and sensitivity will involve optimising the sensor-target subsystem based on a beam dimension and developing hemispheric, or semi-hermetic 2*π*-photodetectors open in the forward direction. The calculations in Fig. 5(a) also corroborate the concave photodetector systems designed elsewhere^35,36^. Hence, one practicable solution to suggest is building an optimised concave photodetector in a honeycombed structure. The benefits from utilising such ultrahigh-precision instruments are expected to ripple out across the scientific community. Further, the position-sensitive detector of fluorescence type, realised by the tailored design process, has a likely impact on applications of wide and far-reaching appeal to the scientific and industrial research alike.

## Electronic supplementary material

Supplementary Information
