# Supplementary material for: Customisable X-ray fluorescence photodetector with submicron sensitivity using a ring array of silicon p-i-n diodes
Source: Sci Rep. 2018 Oct 29;8:15926. doi: 10.1038/s41598-018-33966-y (PMC6206085; doi:10.1038/s41598-018-33966-y)
Supplement: Supplementary file 1 — Supplementary Information [file 41598_2018_33966_MOESM1_ESM.pdf]

Supplementary Information:

Customisable X-ray fluorescence photodetector with submicron sensitivity using a ring array of silicon p-i-n diodes

Phil S. Yoon[1]

[1]P.O. Box 5000, Brookhaven National Laboratory, Upton, NY 11973-5000, USA
[*]corresponding author E-mail: phil.s.yoon@gmail.com

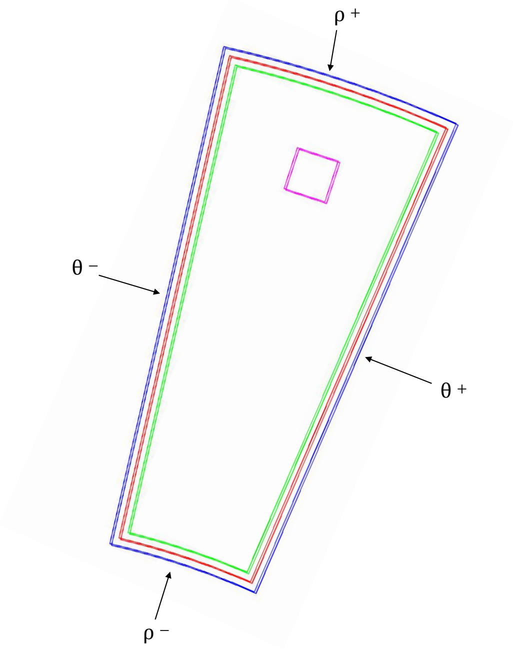

Supplementary Figure 1 | One of the wedge segments consisting of the ring array of *Si* p-i-n diodes; $\rho^{\pm}$ are radial positions measured from the centre of the circular aperture, and $\theta^{\pm}$ are polar angles of the two sidelines.

The solid angle, subtended by one wedge segment at a fluorescent source on target, is derived as follows:

$$\Omega_{ring} = \iint d\,\Omega_{ring} = \int_{\theta_-}^{\theta^+}\int_{\rho^-}^{\rho^+} \frac{z^*\rho\, d\rho\, d\theta}{r^3}$$
$$\mathbf{I}(\rho^+,\theta^+) \;=\; \mathbf{I}(+,+) \;\equiv\; \arctan\left(\frac{\Delta\theta^+(\rho^{*2}-(\rho^*\rho^+)+z^{*2})}{z^*\sqrt{(\Delta\rho^+)^2+(\rho^+\Delta\theta^+)^2+z^{*2}}}\right) \tag{S1}$$

$$\begin{aligned}
\Omega_{ring} &= \Omega_{ring,\,7}(\underbrace{\rho^*,\theta^*,z^*}_{\text{3 beam parameters}}\;;\underbrace{\rho^+,\rho^-,\theta^+,\theta^-}_{\text{4 sensor parameters}})\\
&= Pr(\psi)\cdot\{-\,\mathbf{I}(+,+)\;+\;\mathbf{I}(+,-)\;+\;\mathbf{I}(-,+)\;-\;\mathbf{I}(-,-)\}\\
&= Pr(\psi)\cdot\Bigg\{-\;\arctan\left(\frac{\Delta\theta^+(\rho^{*2}-(\rho^*\rho^+)+z^{*2})}{z^*\sqrt{(\Delta\rho^+)^2+(\rho^+\Delta\theta^+)^2+z^{*2}}}\right)\;+\;\arctan\left(\frac{\Delta\theta^-(\rho^{*2}-(\rho^*\rho^+)+z^{*2})}{z^*\sqrt{(\Delta\rho^+)^2+(\rho^+\Delta\theta^-)^2+z^{*2}}}\right)\\
&+\;\arctan\left(\frac{\Delta\theta^+(\rho^{*2}-(\rho^*\rho^-)+z^{*2})}{z^*\sqrt{(\Delta\rho^-)^2+(\rho^-\Delta\theta^+)^2+z^{*2}}}\right)\;-\;\arctan\left(\frac{\Delta\theta^-(\rho^{*2}-(\rho^*\rho^-)+z^{*2})}{z^*\sqrt{(\Delta\rho^-)^2+(\rho^-\Delta\theta^-)^2+z^{*2}}}\right)\Bigg\},
\end{aligned} \tag{S2}$$

where the projection factor $Pr(\psi)$ is defined to be

$$Pr(\psi) = \cos\psi,\;\; \psi = \arctan\Big((\rho^+ + \rho^-)/(2z^*)\Big)$$

For the case of an on-axis point source, Eqn. (S2) can be reduced to Eqn. (1a) given in the Article.

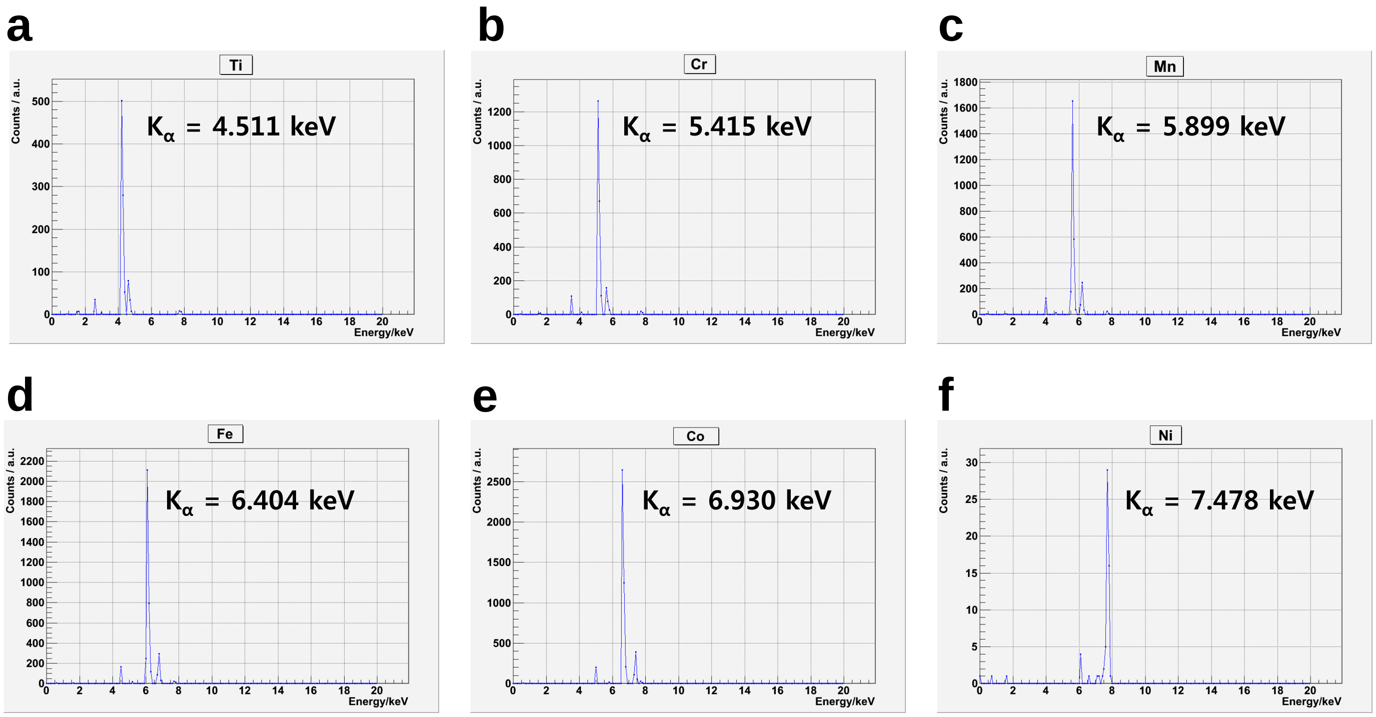

Supplementary Figure 2 | X-ray fluorescence (XRF) spectrum, as a continuous band of energies, emitted upon impingement of 8-keV X-ray photon beams. These energy spectrum were acquired from RÖSI Monte-Carlo simulations with realism, which include photon-beam statistics ($\sim 10^{12}$ photons/sec), the entire detector geometry and assembly in a vacuum at room temperature. The abscissa of each graph is energy in units of keV, whereas the ordinate shows the intensity, or count rate in an arbitrary unit (a.u.). The XRF spectrum in each panel corresponds to each of the six elements to be used as a fluorescing film material: i.e., (**a**) $_{22}$Ti (**b**) $_{24}$Cr (**c**) $_{25}$Mn (**d**) $_{26}$Fe (**e**) $_{27}$Co (**f**) $_{28}$Ni. Inscribed on the background of each plot is the literature value of K_α of each metallic element. The K_α absorption edge has distinctively higher intensity than K_β, peaks of which are accompanied by satellite bumps arising from coherent and incoherent scatters; the intensities of inelastic and elastic scatters are suppressed and minimised, thus turning out to be insubstantial.

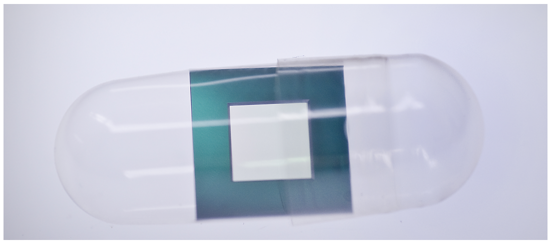

Supplementary Figure 3 | Contained in a capsule is a bare silicon-nitride X-ray window without metallization.
